# Lymphocyte Classification from Hoechst Stained Slides with Deep Learning

**DOI:** 10.3390/cancers14235957

**Published:** 2022-12-01

**Authors:** Jessica Cooper, In Hwa Um, Ognjen Arandjelović, David J. Harrison

**Affiliations:** 1 School of Computer Science, University of St Andrews, St Andrews KY16 9SX, UK; 2 School of Medicine, University of St Andrews, St Andrews KY16 9TF, UK

**Keywords:** deep learning, computer vision, lymphocyte subsets, image classification, imaging

## Abstract

**Simple Summary:**

We train a deep neural network model to identify CD3 expressing cells from Hoechst stained slides only, without the need for costly immunofluorescence. Using interpretability techniques to understand what the model has learned, we find that morphological features in the nuclear chromatin are predictive of CD3 expression.

**Abstract:**

Multiplex immunofluorescence and immunohistochemistry benefit patients by allowing cancer pathologists to identify proteins expressed on the surface of cells. This enables cell classification, better understanding of the tumour microenvironment, and more accurate diagnoses, prognoses, and tailored immunotherapy based on the immune status of individual patients. However, these techniques are expensive. They are time consuming processes which require complex staining and imaging techniques by expert technicians. Hoechst staining is far cheaper and easier to perform, but is not typically used as it binds to DNA rather than to the proteins targeted by immunofluorescence techniques. In this work we show that through the use of deep learning it is possible to identify an immune cell subtype without immunofluorescence. We train a deep convolutional neural network to identify cells expressing the T lymphocyte marker CD3 from Hoechst 33342 stained tissue only. CD3 expressing cells are often used in key prognostic metrics such as assessment of immune cell infiltration, and by identifying them without the need for costly immunofluorescence, we present a promising new approach to cheaper prediction and improvement of patient outcomes. We also show that by using deep learning interpretability techniques, we can gain insight into the previously unknown morphological features which make this possible.

## 1. Introduction

Among patients with cancers of the same stage, clinical outcomes vary widely [1]. This is thought to be in large part due to the complex interaction between tumour cells and the immune response of individual patients, as the proportion, location, and sub-type of lymphocytes present in the tissue has been shown to have important implications for patient prognosis [1,2]. There exist proprietary methods to assess immune cell infiltration, which formally quantify CD3+ and CD8+ T cell lymphocytes both in the centre of tumour and in the invasive margin, as proposed by Galon et al. [3]. Combining their evaluation with T- and B-score (CD8+ T cell and CD20+ B cell) as per Mlecnik et al. had significant predictive power for colorectal cancer patient survival [2,4].

Compared to to the latest guidelines of the American Joint Committee on Cancer/Union for International Cancer Control (AJCC/UICC) tumour-node-metastasis (TNM) classification, immune cell infiltration evaluation alone has shown superior prognostic value in international studies of stage I–IV colon cancer patients. It also has life-saving applications in clinical decision-making [1,3,5,6,7,8,9]. However, in order to identify the cells necessary to calculate these valuable metrics, either multiple immunohistochemistry or multiplexed immunofluorescence are required—both of which are time consuming and expensive protocols [4,10]. Using contemporary equipment, three simultaneous rounds of immunohistochemistry takes around three hours and costs approximately $20 in reagents, whilst multiplex immunofluorescence requires 9 h and the associated reagents cost upward of $70 for a single slide.

In the present work the first step is taken towards decreasing the cost of identifying immune cell subtypes. We show that by using deep learning it is possible to identify CD3 expressing lymphocytes from a common and inexpensive stain. Hoechst and DAPI (popular blue fluorescent, nuclear-specific dyes [11,12,13] staining are far cheaper and easier to perform, costing pennies and requiring just ten minutes per slide. DAPI has better photostability, but since the slides could be imaged immediately in this work Hoechst 33342 is used due to its superior signal-to-noise (genuine DNA stain/autofluorescence) ratio.

Deep learning techniques are increasingly used in digital pathology to assist human experts with a range of diagnostic and prognostic tasks [14,15,16], however, few attempts have been made to tackle the problem of the high-cost of immunofluorescence with machine learning. The main existing approach is virtual staining using GANS (Generative Adversarial Networks) [17,18], in which a model is trained to generate immunofluorescence style stains based unstained (or more cheaply stained) tissue. These virtual stains can then be used for diagnostic or prognostic purposes, either by an automated system or by a human. In this work, we skip the virtual staining step and go straight from Hoechst stained image to classification. For image classification tasks CNNs (Convolutional Neural Networks) are most widely used [19], as we do herein.

The novelty of our approach is therefore twofold: firstly, we show that it is possible to identify CD3 expressing lymphocytes from Hoechst stained tissue; and secondly, we do this without using the interim method of virtual staining.

Our methodology is as follows: we image each tissue section with both Hoechst and immunofluorescence stains; identify which cells express CD3 using an intensity-based classifier on the immunofluorescence images; and use those classifications to label the same cells in the Hoechst-stained images. We then use these Hoechst-image/immunofluorescence-classification pairs to train a deep neural network to classify CD3 expressing cells, using the Hoechst images only as input. In this way, we force the network to find patterns in the Hoechst-stained cells that correspond to the correct immunofluorescence labels, without ever being exposed to the actual immunofluorescence images.

## 2. Materials and Methods

The data in this study comprised thirty WSIs taken from cancer biopsies. The slides were provided by NHS Lothian and were deidentified to preserve patients’ anonymity. The thirty slides were randomly selected from three larger cohorts of consenting patients, and each slide was from a different patient. Ten slides were from lung cancer patients, ten from colon cancer patients, and ten from kidney cancer patients. These were imaged using Hoechst 33342, and also using immunofluorescence targeting CD3 expressing immune cells, with a Zeiss Zen Axioscan scanner. An established intensity based classification technique [20] was then used to identify CD3 expression and label these cells in the immunofluoresence images, the results of which were quality controlled by direct visual inspection to ensure label accuracy. A cell classification dataset was then generated by extracting individual images of each cell and pairing them with the immunofluorescence-generated labels.

### 2.1. Immunofluorescence (IF) Protocol

Leica BOND RX automated immunostainer (Leica Microsystems, Milton Keynes, UK) was utilised to perform mIF. The sections were dewaxed at 72 ${}^{\circ }$C using BOND dewax solution (Leica, AR9222) and rehydrated in absolute alcohol and deionised water, respectively. The sections were treated with BOND epitope retrieval 1 (ER1) buffer (Leica, AR9961) for 20 min at 100 ${}^{\circ }$C to unmask the epitopes. The endogenous peroxidase was blocked with peroxide block (Leica, DS9800), followed by serum free protein block (Agilent, x090930-2). Then the sections were incubated with the primary antibody (CD3, Agilent, A045229-2, 1:70 dilution), followed by biotinylated anti-rabbit secondary antibodies (Thermo Fisher, 65-6140), which was visualised by Alexa flour 750 conjugated streptavidin (Thermo Fisher, S21384). Cell nuclei were counterstained by Hoechst 33342 (Thermo Fisher, H3570, 1:100) and the sections were mounted with prolong gold antifade mountant (Thermo Fisher, P36930).

### 2.2. Image Acquisition and Analysis

Zeiss Axioscan z1 was used to capture fluorescent images at 20× object magnification. Two different fluorescent channels, Hoechst3334 and AF750 were simultaneously used to capture individual channel images under 20× object magnification. The exposure time of the channels were 8 and 800 ms, respectively. The image was generated in CZI (Carl Zeiss Image) format. The fluorescent images were opened in QuPath v.0.2.3 [21]. StarDist [22] was used to segment cell nuclei using StarDist2D builder. The probability threshold of cell detection, pixel size and the cell expansion was 0.6, 0.2270 and 1.0, respectively. The object classifier was utilised to classify CD3 cells with an intensity threshold of 2200 in the AF750 channel.

**Figure 1:**
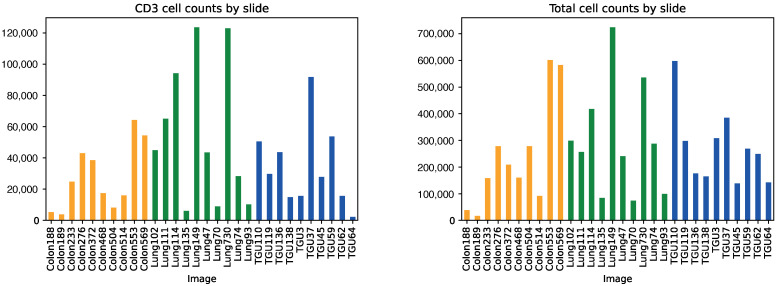
Cell counts per slide. Colon and lung slides (shown in yellow and green respectively) formed the training and validation set. Kidney cancer slides (shown in blue) exclusively formed the holdout test set.

The total number of labelled cells present across all slides was 146,883,654. As shown in Figure 1 these were unequally distributed across the slides, ranging from 16,991 to 723,458 labelled cells per slide. Of these cells, only 21,018,870 expressed CD3—just 14.3%—and these too were unequally distributed, ranging from 2166 to 122,929.

As shown in Table 1, the nucleus and cell measurement features varied to a large degree. To test whether these simple morphological features alone had predictive power, we attempted to train a number of different statistical and neural network binary classification models to discriminate between CD3 cells and an equal number of randomly selected other cells, based on nucleus area, length, circularity, maximum and minimum diameter, and solidity. These included a simple linear regression model, and five- and ten-layer neural networks with ReLU activations. These were trained on a variety of hyperparameters using grid search, but it was not possible to reach better than chance accuracy, as there is not enough information in the cell measurements alone to identify CD3 expressing cells. We then turned to more complex convolutional neural networks to enable direct representation learning from images. Herein we describe the success found using a standard wide resnet50. (Other architectures of similar type and size performed comparably).

To create a balanced dataset, from the Hoechst-stained slides all CD3 expressing cells and an equal number of randomly selected non-CD3 expressing cells were exported at full resolution. Individual cells were isolated by masking out the background such that each sample contained one cell only. Each of these single-cell images was of dimension 64×64. Each cell image was normalised individually prior to training. Normalisation was used instead of standardisation to account for variability in pixel value range between slides. From the thirty slides, all ten kidney cancer slides were held out as test set. Two slides were selected randomly from each of the remaining lung and colon cancer cohorts for use in validation, and the remaining eight from each were used for training. Due to differing numbers of patches available per slide, this provided a total of 1,159,562, 485,206 and 690,662 total cell images in the training, validation and test set, respectively.

### 2.3. Model Architecture and Training

All computation was performed using eight NVIDIA Tesla V100 GPUs.

The classification model (a standard torchvision WideResnet50) was trained for up to 100 epochs using Adam optimisation [23], with a batch size of 512 and a learning rate of 0.000001. Early stopping was performed to limit overfit, with training halted if no decrease in validation loss was observed for 10 epochs—this resulted in the model being trained for 34 epochs in total. In order to directly optimise for a balance of precision and recall, we used the F1 score as the loss function, such that:

(1) $$L\left(\rho ,\tau \right)=1-\frac{1}{C}(\sum_{c=0}^{C}\frac{2\frac{\tau_{c}\rho_{c}+\epsilon }{\tau_{c}\rho_{c}+\left(1-\tau_{c}\right)\rho_{c}+\epsilon }\frac{\tau_{c}\rho_{c}+\epsilon }{\tau_{c}\rho_{c}+\tau_{c}\left(1-\rho_{c}\right)+\epsilon }}{\frac{\tau_{c}\rho_{c}+\epsilon }{\tau_{c}\rho_{c}+\left(\left(1-\tau_{c}\right)\rho_{c}\right)+\epsilon }+\frac{\tau_{c}\rho_{c}+\epsilon }{\tau_{c}\rho_{c}+\tau_{c}\left(1-\rho_{c}\right)+\epsilon }})$$

is minimised, where τ is the target class in one-hot form (e.g., a CD3 expressing cell label is encoded as [0,1]) and ρ is the softmaxed model output. Empirically we found that using this F1 loss instead of the more usual cross entropy resulted in an increase in accuracy of around 7%. This protocol was designed after significant experimentation, considering a range of architectures and hyperparameters. Both other custom built models and pretrained ones available in the Pytorch model zoo provided either no significant increase proportional to computation cost, or a decrease in model performance.

## 3. Results

**Figure 2:**
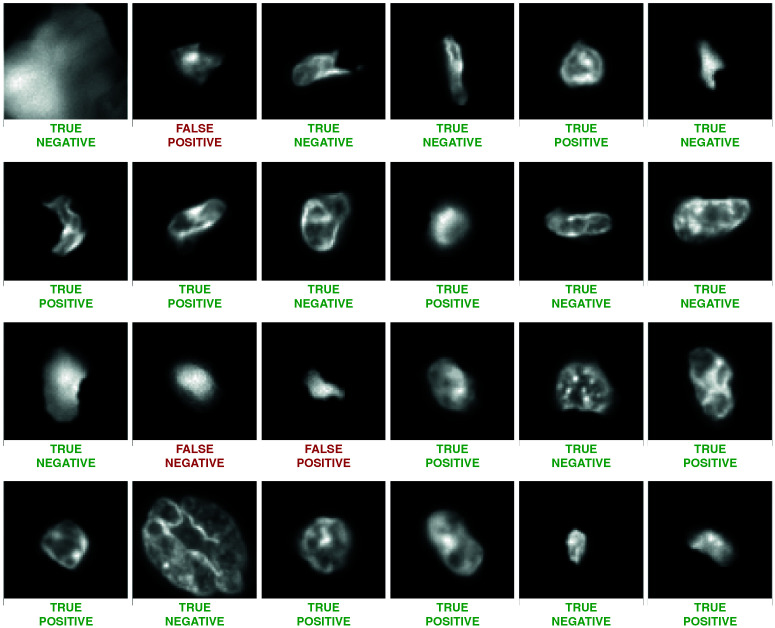
Example cell samples and model predictions from the test set.

**Figure 3:**
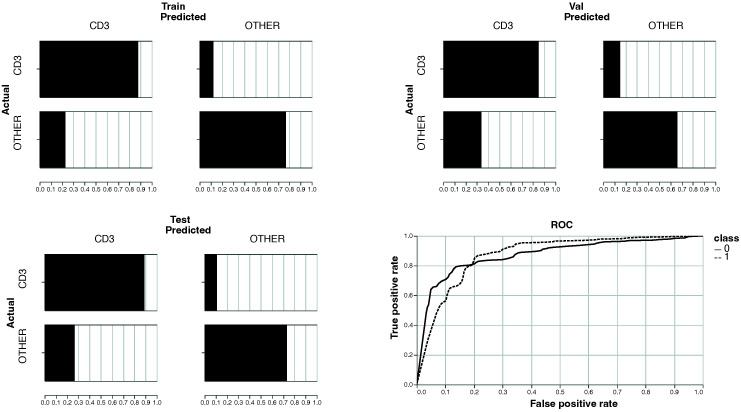
Confusion matrices for training, validation and test sets, along with Receiver Operator Characteristic (ROC) curve from test set only.

Table 2 shows the performance of the model according to these metrics. The model achieved over 80% precision, recall and F1 score on the test set, showing excellent generalisation to unseen slides. Moreover, since the test slides were from kidney cancer slides and the training and validation sets from only lung and colon cancer slides, this shows that the ability to identify CD3 expressing cells from morphological features made visible by Hoechst staining is not limited to lung and colon cancer patients, and can be generalised from them to patients with other cancers. Figure 2 shows a number of example cells from the test set, along with their ground-truth classification and the model’s prediction. Figure 3 shows the confusion matrices for training, validation and test sets, demonstrating robust and generalisable classification ability with little evidence of overfit.

**Figure 4:**
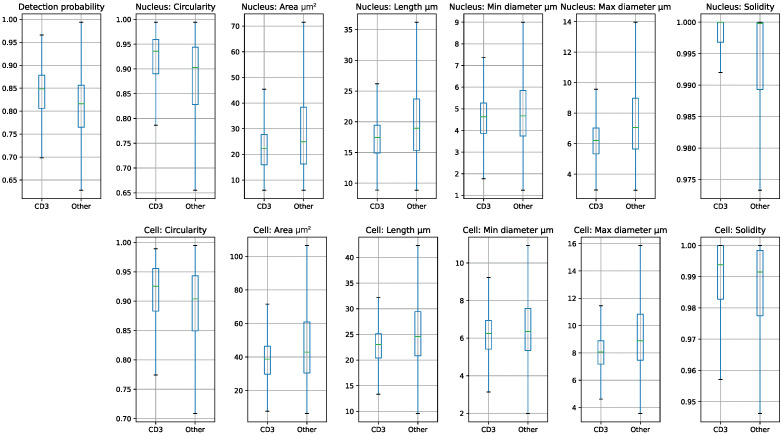
Cell feature box plots for CD3-expressing and ’Other’ cells in the dataset.

Inspection of the dataset and statistics in the previous section (see Figure 4) shows that CD3 expressing cells are on average smaller, and exhibit a higher degree of nuclear solidity than other cells in Hoechst imaging. Since each cell image was individually normalised prior to training and inference, any relative difference in intensity between cells of different types would be mitigated to a large extent. However, most of these differences in distribution would remain even after normalisation, so to explore whether this higher solidity and difference in size is used by the classification model in preference to morphological features, training and validation were repeated using the same slides at 2× lower magnification level. This preserves shape, relative size and relative intensity but obscures fine-grained features at a cellular level. On this training data the model performance on validation was far lower, indicating that small features visible at the highest magnification level were necessary to achieve these results.

## 4. Discussion

In this section we employ Hierarchical Perturbation (HiPe) [24] and standard iterative perturbation [25] to understand how the model is able to identify CD3 expressing lymphocytes. These methods are widely used for deep learning interpretability as they offer intuitive visual interpretations of which regions in the input were more or less important in determining the model’s output. Both work by perturbing regions of the input and using the change in the model’s output due to that perturbation to build up a saliency map. Iterative perturbation does this sequentially, by passing a perturbation kernel of fixed size k×k over the input. HiPe does this more dynamically, beginning by perturbing large, overlapping regions and inspecting the relative difference in saliency between those regions. All regions of the saliency map which exceed a threshold (the mid-range, in the standard implementation) are split into smaller overlapping regions, which are then each perturbed, and the saliency map and threshold updated in turn until either the minimum perturbation size is reached, or no region remains above the saliency threshold. HiPe is typically much faster than standard iterative perturbation, as by ignoring regions of relative unimportance the number of operations required is reduced. It also has the benefit of requiring no kernel size to be specified, as (unlike iterative perturbation) it is capable of identifying salient features of any dimension. HiPe was used in preference to other input saliency based explanatory techniques as it is much quicker than similar perturbation-based saliency methods, and is more precise than gradient-based methods which are often indistinct.

**Figure 5:**
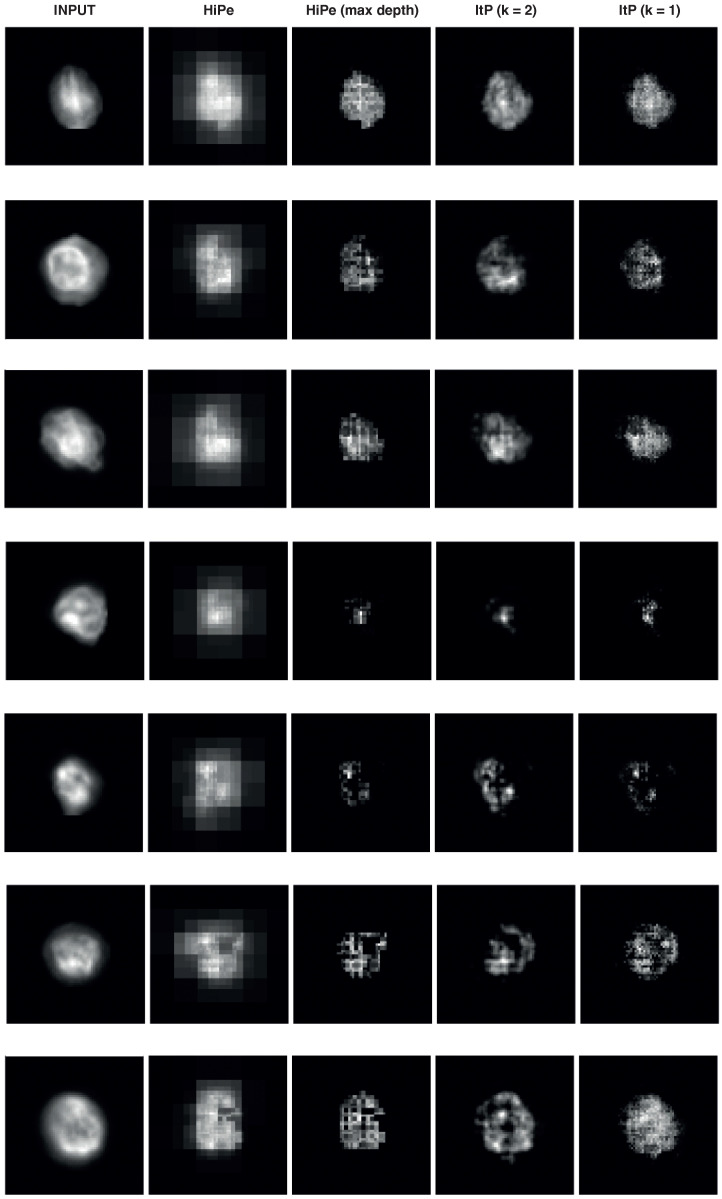
Saliency maps generated by Hierarchical Perturbation (HiPe) on CD3 expressing cells, at both all depths and maximum depth only, plus saliency maps generated by standard Iterative Perturbation (ItP) with kernel sizes of 1 and 2.

Hierarchical Perturbation was used to generate saliency maps for CD3 classified cells as shown in Figure 5.

The standard implementation of HiPe was used on the softmaxed output of the model, with “fade” perturbation, such that perturbed portions are replaced with zero input. Additionally, the HiPe saliency maps for each step of the process (i.e., at each kernel size) were retained in order to isolate the smallest salient features. Input saliency based methods like HiPe explicitly show which areas of the input image were more or less important in determining the output for each class. For comparison we also include standard iterative perturbation saliency maps with kernel sizes of 2×2 and 1×1, as the extra computational cost is not too onerous for these small cell images. Inspection of the saliency maps shown in Figure 5 shows that larger salient regions comprised the cells themselves, as would be expected—but more interestingly, that the most salient regions were much smaller, appearing to cluster in the nuclei of the salient cells. We note also that the outer edges of cells do not appear salient at all, indicating that the model did not learn to use the circumference, circularity or size of the cells to make predictions, as suspected based on our previous attempt to train a tabular classifier model from these measurements. This supports the hypothesis that the model is using morphological features of the chromatin made visible by Hoechst 33342 staining to perform the classification.

## 5. Conclusions

In this work we demonstrate that it is possible to identify cells expressing CD3 using Hoechst staining only. Moreover, we show that with interpretability techniques, neural networks can become valuable tools for discovery as well as for automation: using saliency mapping we visualise which features in the input the model is using to make correct classifications, and find that these saliency maps highlight the nuclear chromatin within the cells, indicating that the chromatin texture and morphology made visible by Hoechst staining is predictive of CD3 expression.

Future work will include exploring semi-supervised and unsupervised approaches to classification via clustering to reduce labelling burden when training new models, alongside extending and applying this approach to other cancers and proteins. It is our hope that the application of proven prognostic metrics (such as immune cell infiltration evaluation) to slides labelled using our method will drastically reduce the cost of immune profiling and thereby allow more patients to benefit.

## Figures and Tables

**Table 1:** Cell and nucleus statistics for all data.

	Mean	Std	Min	25%	50%	75%	Max
Detection probability	0.81	0.07	0.60	0.77	0.82	0.86	1.00
Nucleus: Area µm${}^{2}$	29.70	21.23	6.00	16.20	24.33	35.83	1084.42
Nucleus: Length µm	19.74	6.40	8.84	15.28	18.58	22.84	252.07
Nucleus: Circularity	0.88	0.10	0.15	0.84	0.91	0.95	0.99
Nucleus: Solidity	0.99	0.03	0.32	0.99	1.00	1.00	1.00
Nucleus: Max diameter µm	7.39	2.57	2.95	5.59	6.86	8.65	60.04
Nucleus: Min diameter µm	4.90	1.65	1.23	3.76	4.67	5.71	47.91
Cell: Area µm${}^{2}$	48.21	26.95	6.28	30.36	41.97	57.66	1200.90
Cell: Length µm	25.35	6.52	9.56	20.76	24.23	28.58	258.87
Cell: Circularity	0.89	0.08	0.16	0.85	0.91	0.95	0.99
Cell: Solidity	0.98	0.03	0.39	0.98	0.99	1.00	1.00
Cell: Max diameter µm	9.22	2.58	3.56	7.42	8.70	10.50	62.15
Cell: Min diameter µm	6.56	1.71	1.84	5.35	6.33	7.43	48.78
Nucleus/Cell area ratio	0.58	0.09	0.27	0.52	0.58	0.64	1.00

**Table 2:** Classification performance on training, validation and test data.

	F1	Precision	Recall	Accuracy
Training	0.802	0.806	0.803	0.802
Validation	0.773	0793	0.776	0.776
Test	0.805	0.807	0.805	0.805

## Data Availability

Code is available here: https://github.com/jessicamarycooper/ICAIRD. Data is available from the authors upon request.
